# HrcU and HrpP are pathogenicity factors in the fire blight pathogen *Erwinia amylovora* required for the type III secretion of DspA/E

**DOI:** 10.1186/s12866-016-0702-y

**Published:** 2016-05-20

**Authors:** R. Ryan McNally, Quan Zeng, George W. Sundin

**Affiliations:** Department of Plant Pathology, University of Minnesota, St. Paul, MN 55108 USA; Department of Plant, Soil, and Microbial Sciences, Michigan State University, East Lansing, MI 48824 USA; Department of Plant Pathology and Ecology, Connecticut Agricultural Experiment Station, New Haven, CT 06504 USA

**Keywords:** Type III secretion system, Secretion hierarchy, Substrate specificity, *Erwinia amylovora*, HrcU, HrpP

## Abstract

**Background:**

Many Gram-negative bacterial pathogens mediate host-microbe interactions via utilization of the type III secretion (T3S) system. The T3S system is a complex molecular machine consisting of more than 20 proteins. Collectively, these proteins translocate effectors across extracellular space and into the host cytoplasm. Successful translocation requires timely synthesis and allocation of both structural and secreted T3S proteins. Based on amino acid conservation in animal pathogenic bacteria, HrcU and HrpP were examined for their roles in regulation of T3S hierarchy.

**Results:**

Both HrcU and HrpP were shown to be required for disease development in an immature pear infection model and respective mutants were unable to induce a hypersensitive response in tobacco. Using in vitro western blot analyses, both proteins were also shown to be required for the secretion of DspA/E, a type 3 effector and an important pathogenicity factor. Via yeast-two hybridization (Y2H), HrpP and HrcU were revealed to exhibit protein-protein binding. Finally, all HrcU and HrpP phenotypes identified were shown to be dependent on a conserved amino acid motif in the cytoplasmic tail of HrcU.

**Conclusions:**

Collectively, these data demonstrate roles for HrcU and HrpP in regulating T3S and represent the first attempt in understanding T3S heirarchy in *E. amylovora*.

**Electronic supplementary material:**

The online version of this article (doi:10.1186/s12866-016-0702-y) contains supplementary material, which is available to authorized users.

## Background

The type III secretion (T3S) system is a common feature of Gram-negative bacterial pathogens. The T3S system functions to facilitate the translocation of bacterial effector proteins into eukaryotic host cells where they suppress host defense responses, facilitate colonization, and promote disease development [1]. Consequently, T3S has been the focus of intensive research in both animal and plant pathosystems.

The T3S system is a complex proteinaceous machine consisting of more than 20 components. Because the successful translocation of bacterial effectors necessitates a functioning multipartite machine, the production of structural and secreted T3S system components has been assumed to be hierarchical. Recent analyses have confirmed the hierarchical nature of T3S in a few animal and plant pathogens [2–7]. Characterization of this hierarchy has revealed multiple substrate classes. Early substrates are involved in pilus formation while late substrates, like effectors, are secreted after the assembly of a complete T3S system.

An array of factors has been implicated in regulating T3S system hierarchy [8]. Predominately featured are two protein groups 1) YscU/FlhB proteins and 2) YscP/FliK-like proteins. YscU/FlhB proteins include YscU, a T3S protein from *Yersinia* spp. and FlhB, a flagellar protein from *Salmonella* spp., which represent the most characterized regulators of T3S hierarchy [2–5, 9]. YscU/FlhB proteins exhibit four N-terminal transmembrane domains that play a structural role in the inner membrane export apparatus of the T3S system basal body [10, 11]. T3S is completely abolished in *yscU/flhB* null mutants [5, 12, 13]. The C-termini of YscU/FlhB proteins, however, encode a characteristic cytoplasmic domain involved in regulating T3S system hierarchy [3, 5, 9, 12, 14]. This domain is required for conformational changes via autoproteolytic cleavage at an Asp-Pro-Thr-His (NPTH) motif [4, 9, 15]. The NPTH motif is conserved in all YscU/FlhB homologs and point mutations in the NPTH motif are frequently associated with phenotypes including 1) avirulence, 2) loss of protein-protein interactions and 3) loss of secretion heirarchy [4, 13]. Due to the location of YscU/FlhB proteins at the basal body-cytoplasm interface and due to their role in regulating T3S hierarchy, YscU/FlhB proteins display numerous protein-protein interactions [3, 5, 16, 17]. For example, HrcU from *X. campestris* has been demonstrated to interact with at least seven other T3S proteins [16, 18–20]. Among these HrcU-interacting proteins are YscP/FliK-like proteins. YscP/FliK-like proteins differ from the YscU/FlhB protein in that they share little amino acid sequence conservation. They are hydrophobic, globular and contain a Pro-X-Leu-Gly C-terminal motif [21]. Mutations affecting YscP/FliK-like proteins frequently compromise the ability of T3S systems to change substrate specificity during hierarchical T3S and consequently are termed T3S substrate specificity switches (T3S4) [8]. T3S4 mutant phenotypes include 1) reduced secretion of late substrates, 2) increased filament length, and sometimes 3) increased secretion of early substrates [6, 16, 22–25]. YscP from *Yersinia* spp., FliK from flagella, and HpaC from *Xanthomonas campestris* all represent T3S4 proteins. Both FliK and HpaC have been demonstrated to directly bind the cytoplasmic domains of their cognate YscU/FlhB proteins, and phenotypes associated with NPTH domain mutations are attributed to loss of protein-protein interaction with T3S4 proteins [3, 4, 9, 16, 25].

The Gram-negative plant pathogenic bacterium *Erwinia amylovora* is the causative agent of fire blight, a disease of rosaceous species including apple and pear. Disease development by *E. amylovora* requires a functioning T3S system [26]. In *E. amylovora*, the T3S system is known to secrete at least 12 proteins including the harpins HrpN and HrpW as well as the effector DspA/E (hereafter termed DspE), a pathogenicity factor [27–29]. To date, little is known about how secretion hierarchy is regulated in *E. amylovora*. While HrpJ is required for secretion of translocators HrpN and HrpW, nothing is known about how *E. amylovora* regulates the substrate specificity of DspE, the most important component of the T3S system for fire blight disease development [29]. In *E. amylovora*, YscU/FlhB and TS34 proteins are represented by HrcU (EAM_2905) and HrpP (EAM_2900), respectively. Here, HrcU and HrpP are explored for roles in T3S system regulation in *E. amylovora*.

## Results

### HrcU exhibits a conserved NPTH motif required for pathogenicity in *E. amylovora*

**Fig. 1:**
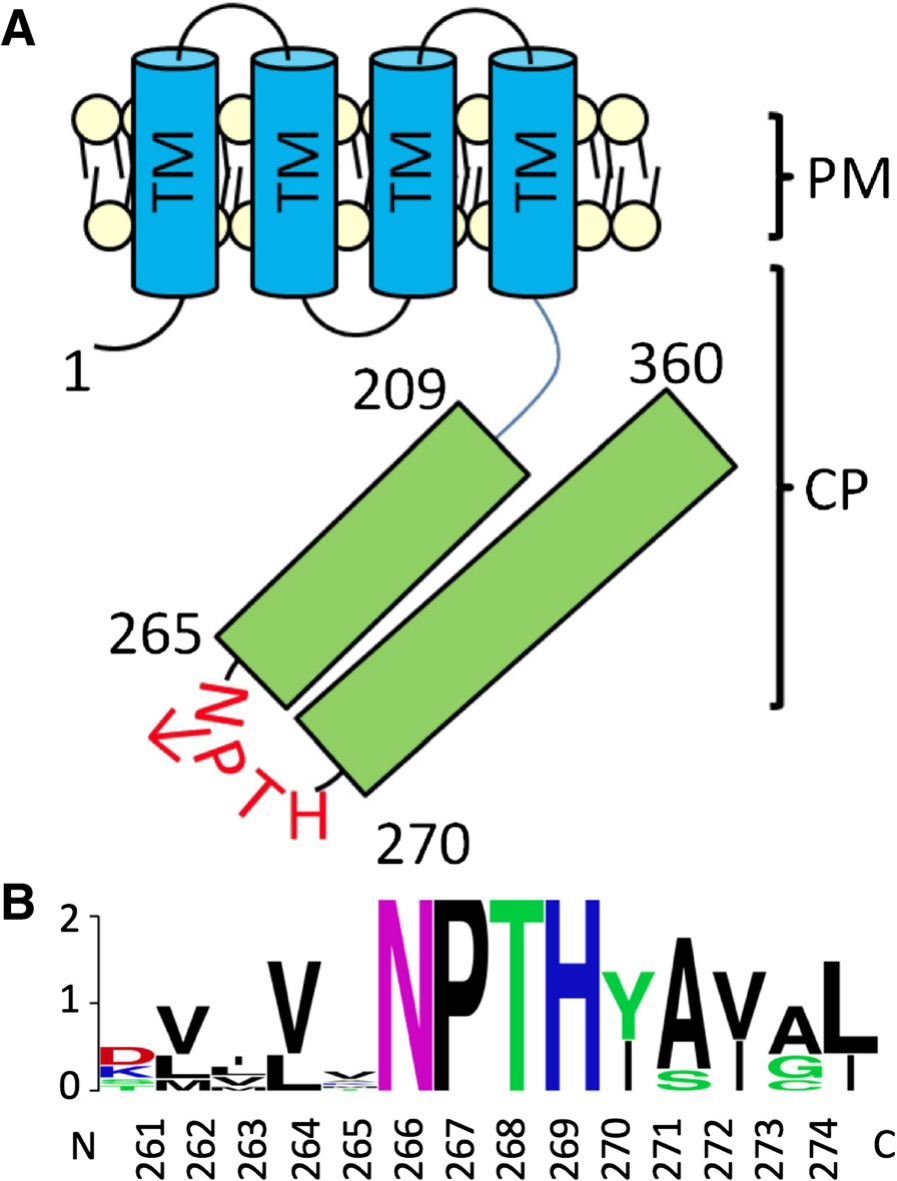
HrcU in *Erwinia amylovora*. **a** Schematic representation of HrcU domain organization. Letters indicate predicted transmembrane domains (TM), the plasmamembrane (PM) and the cytoplasm (CP). Numbers denote amino acid positions based on the genome sequence of *E. amylovora* ATCC 49946 (NCBI NC_013971). The NPTH motif is labeled in red and the arrow represents the site of cleavage and conformational change reported in homologous proteins. **b** T-coffee multiple sequence alignment of C-terminal NPTH motif in HrcU homologs. Weblogo was used to visualize an alignment of HrcU homologs from animal pathogens *Yersinia enterocolitica*, *Shigella flexneri* and *Escherichia coli*, plant pathogens *Erwinia amylovora*, *Xanthomonas campestris* and *Pseudomonas syringae* as well as a flagellar homolog from *Salmonella enteric*. NPTH is conserved in all HrcU homologs

The NPTH motif in YscU/FlhB proteins is the site of autoproteolytic cleavage and conformational change required for protein function [4, 15]. This NPTH motif is conserved in all known YscU/FlhB proteins [3, 5, 30]. Bioinformatic analysis of HrcU from *E. amylovora* using a dense alignment surface algorithm predicted that, like YscU/FlhB homologs, HrcU encodes four transmembrane domains as well as a cytoplasmic C-terminal tail (Fig. 1a) [10, 31]. Using T-Coffee multiple alignment software, the amino acid sequence of HrcU was compared to multiple homologs in T3S systems of plant and animal bacterial pathogens as well as in the flagellum (Table 1) [32]. The *E. amylovora* HrcU NPTH motif (HrcU_NPTH_) was found to be conserved in *E. amylovora* and in all analyzed homologs (Fig. 1b).

**Table 1:** YscU/FlhB family proteins used for sequence alignment

Protein	Accession	Bacterium
YscU	NC_004564.1	*Yersinia enterocolitica* A127/90
FlhB	NC_021176.1	*Salmonella enteric* Ty21a
Spa40	AY206439.1	*Shigella flexneri*
EscU	AE005174.2	*Escherichia coli* O157:H7
HrcU	NC_013971.1	*Erwinia amylovora* ATCC 49946
HrcU	NC_007508.1	*Xanthomonas campestris* pv. *vesicatoria* str. 85-10
HrcU	NC_004578.1	*Pseudomonas syringae* pv. *tomato* str. DC3000

**Fig. 2:**
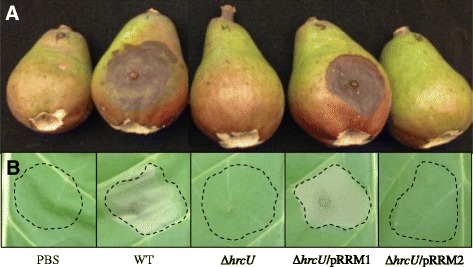
Phenotypic characterization of HrcU-related mutant strains in Ea1189. WT Ea1189, Ea1189∆*hrcU* and Ea1189∆*hrcU* stains expressing native HrcU from pRRM1 or HrcU_N266A_ from pRRM2 were inoculated into **a** immature pear fruits and **b** *Nicotiana benthamiana*. Pear fruit necrosis was recorded 6 days post inoculation while the hypersensistive response in *N. benthamiana* was observed 16 h post inoculation. Ea1189∆*hrcU* was non-pathogenic and unable to elicit a hypersensitive response while pRRM2 was unable to complement the *hrcU* null mutation

To determine the role of HrcU in disease development, a chromosomal deletion of *hrcU* was created in *E. amylovora* Ea1189. Ea1189∆*hrcU* was confirmed to be nonpathogenic due to a lack of symptom development 6 days post inoculation (dpi) in an immature pear infection model (Fig. 2a). *In trans* expression of *hrcU* via the plasmid pRRM1 was able to successfully complement the mutant strain restoring full virulence to Ea1189∆*hrcU* (Fig. 2a).

To ascertain the importance of HrcU_NPTH_ in *E. amylovora*, HrcU was subjected to site-directed mutagenesis. The asparagine residue of the NPTH motif is required for YscU/FlhB protein function in assayed homologs [4, 5, 17]. Consequently, the conserved asparagine residue located at position 266 in the amino acid sequence of HrcU was mutated to encode a codon corresponding to alanine. This *hrcU* mutant allele (HrcU_N266A_) was cloned into an expression vector creating pRRM2 [33].

To determine the role of HrcU_N266A_ in host-microbe interactions, Ea1189∆*hrcU*/pRRM1 and Ea1189∆*hrcU*/pRRM2 were inoculated into immature pear fruits. While plasmid-borne *hrcU* was able to re-establish wild type (WT) virulence levels to Ea1189∆*hrcU*, Ea1189∆*hrcU*/pRRM2 was unable to restore pathogenicity 6 dpi in immature pear fruits (Fig. 2a). This indicates that HrcU_NPTH_ is required for HrcU function and that HrcU_NPTH_ is necessary to mediate compatible host interactions.

### HrcU_NPTH_ is required for the elicitation of the hypersensitive response

The hypersensitive response (HR) is a hallmark of incompatible plant-microbe interactions. The HR is characterized by rapid, localized programmed cell-death in response to pathogen-associated proteins frequently represented by T3S system substrates. HR elicitation in *E. amylovora* requires a functional T3S system [34]. *E. amylovora* Ea1189 strains were inoculated into *Nicotiana benthamiana* mesophyll tissue and, 16 h post inoculation (hpi), results revealed that *E. amylovora* Ea1189 requires HrcU, and specifically HrcU_NPTH_**,** for HR development (Fig. 2b). While WT Ea1189 and complemented Ea1189∆*hrcU*/pRRM1 induced robust HR symptoms in *N. benthamiana*, Ea1189∆*hrcU* and Ea1189∆*hrcU* expressing HrcU_N266A_ failed to trigger an incompatible defense response (Fig. 2b). As the HR in response to *E. amylovora* infection requires T3S, these results suggest that the inability of HrcU_N266A_ to complement Ea1189∆*hrcU* is due to the disrupted function of HrcU_NPTH_ in mediating T3S.

### HrpP is required for pathogenicity and hypersensitive response induction

**Fig. 3:**
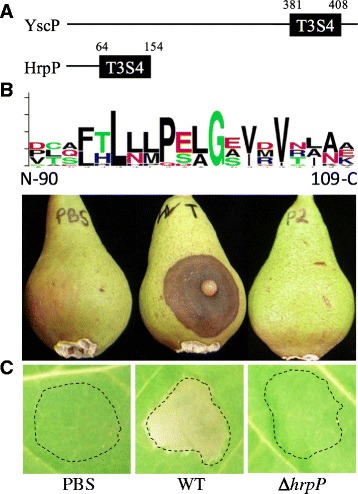
Bioinformatic and phenotypic analyses of HrpP from *E. amylovora*. **a** Schematic representation of T3S4 domain protein alignment from animal and plant pathogenic bacteria. The length of the lines and boxes represent the actual sizes of the protein and domain. Numbers indicate amino acid position. HrpP in *E. amylovora* is markedly smaller than T3S4 proteins in animal bacterial pathogens and the flagellum. **b** Visualization of T3S4 protein sequence alignments with Weblogo software demonstrates that HrpP exhibits a conserved P-X-L-G motif characteristic of T3S4 proteins. **c** WT Ea1189 and Ea1189∆*hrpP* inoculated into immature pear fruits and *N. benthamiana*. Pear fruit necrosis was recorded 6 days post inoculation while the hypersensistive response in *N. benthamiana* was observed 16 h post inoculation. HrpP is a pathogenicity factor required for HR elicitation

In YscU/FlhB proteins, the NPTH motif is required for the regulation of T3S hierarchy [3–5, 12]. T3S system hierarchy regulation is mediated via direct and indirect interactions with T3S4 proteins [3, 4, 16, 25]. In *E. amylovora*, HrpP (EAM_2900) is a predicted T3S4 protein. Bioinformatic analyses of the HrpP amino acid sequence are in accordance with previous observations that T3S4 proteins are poorly conserved between species and that, in bacterial plant pathogens, T3S4 proteins are N-terminally truncated relative to homologs in animal pathogenic bacteria and the flagellum (Fig 3a) [8]. Beginning at amino acid position 98 though, HrpP does exhibit a modified Pro-X-Leu-Gly motif that is characteristic of T3S4 with alanine replacing leucine at position 100 (Pro-Glu-Ala-Gly) (Fig 3b) [35].

To establish the role of HrpP in mediating plant-microbe interactions, a chromosomal deletion of HrpP was synthesized, and relevant strains were inoculated into host and non-host plant species. Like Ea1189∆*hrcU* strains expressing HrcU_N266A_, Ea1189∆*hrpP* was nonpathogenic 6 dpi in immature pear fruit and unable to elicit a HR in *N. benthamiana* (Fig 3c).

### HrcU and HrpP interact in *E. amylovora*

While not all T3S4 proteins have been observed to interact directly with YscU/FlhB counterparts, direct interactions have been recorded between the T3S4 proteins FliK and HpaC [3, 16]. To explore the possibility of HrpP interactions with HrcU in *E. amylovora*, *hrpP* and *hrcU* constructs were cloned into Y2H vectors and assayed in *Saccharomyces cerevisiae* AH109 via survival on minimal medium and α-galactosidase activity. In the Y2H assay, *hrcU* alleles featuring the HrcU_N266A_ point mutation were included along with N-terminal *hrcU* deletions (HrcU-CT). HrcU-CT constructs were included due to reported transmembrane domain interference with protein-protein interactivity in homologous YscU/FlhB proteins [3, 16]. Alongside HrpP, the T3S protein HrpJ was also screened for the ability to interact with HrcU in yeast as HrpJ is a demonstrated regulator of T3S system hierarchy in *E. amylovora* and homologs are required for late substrate secretion due to their roles as T3S inner rod proteins [29]. Another example of this occurs in *Pseudomonas syringae* where HrpJ functions within the bacterial cell to control secretion of translocator proteins such as the harpins HrpZ1 and HrpW1 [36].

**Fig. 4:**
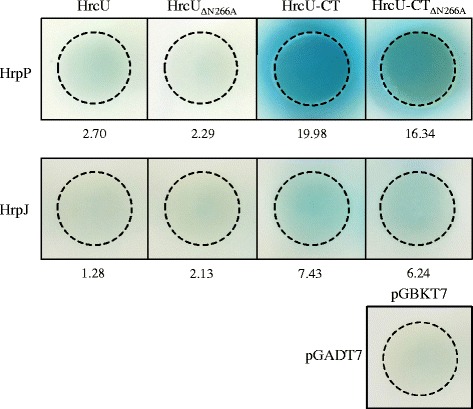
HrcU yeast two-hybrid interaction assays (Y2H). Native HrcU, HrcU_N266A_ and C-terminal (CT) truncations of both proteins were cloned into the bait vector pGBKT7. HrpJ and HrpP were expressed from the prey vector pGADT7. Blue coloration indicates the strength of protein–protein interactions. All Y2H interactions were quantified relative to an empty-vector control using ImageJ software and tested for significance using Kendall rank correlation coefficient τ tests. Both full-length and truncated HrcU_N266A_ exhibited impaired interactions with HrpP and HrpJ relative to native HrcU constructs

In all cases, full-length HrcU encoding N-terminal transmembrane domains were unable to interact with either HrpP or HrpJ (Fig 4). HrpJ exhibited a very weak interaction with both HrcU-CT and HrcU-CT_N266A_ (Fig 4). Conversely, HrpP interacted strongly with HrcU-CT in Y2H experiments (Fig 4). While the HrcU_NPTH_ motif was not absolutely required for interactions with HrpP, HrcU-CT_N266A_ displayed less α-galactosidase activity in the presence of HrpP than did HrcU-CT (Fig 4). This indicates that HrpP does interact with HrcU and that HrcU_NPTH_-mediated conformational changes in HrcU affect HrpP binding in Y2H assays. All qualitative Y2H results were assessed quantitatively using image analysis software ImageJ and shown to be statistically significant.

### HrcU_NPTH_ and HrpP are required for the secretion of DspE

**Fig. 5:**
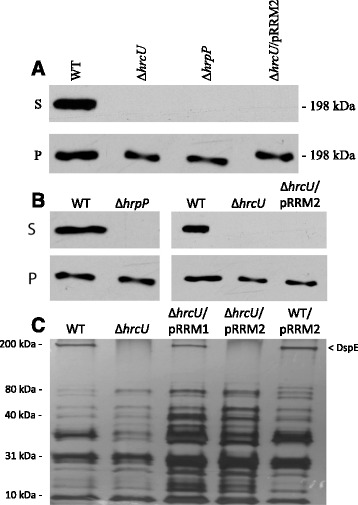
DspE secretion in Ea1189 strains. **a** Composite image of in vitro secretion of DspE-cyaA fusion proteins in minimal medium was visualized via western blot assay using an anti-CyaA antibody. DspE localization was detected in both the culture supernatant (S) and the cell pellet (P). Native HrcU, HrpP and the HrcU_NPTH_ domain were required for DspE secretion as Ea1189∆*hrcU*/pRRM2 expressing HrcU_N266A_ was unable to complement DspE secretion into the supernatant. HrcU and HrpP did not affect the production of DspE in the cell pellet. **b** Separate images composing (**a**). **c** T3S secretome in Ea1189 strains. All strains were cultured in minimal medium and after processing, were separated via one-dimensional SDS-PAGE and stained with silver nitrate. DspE is represented by a band corresponding to ~200 kDa and marked with an arrow. Both WT Ea1189 and Ea1189Δ*hrcU*/pRRM1 secrete DspE in vitro. Ea1189Δ*hrcU* and Ea1189Δ*hrcU*/pRRM2 cannot secret DspE indicating that HrcU_N266A_ is required for DspE secretion. WT Ea1189 producing HrcU_N266A_ does not exhibit a dominant negative effect on DspE secretion

The T3S system effector DspE is a pathogenicity factor of *E. amylovora* and the translocation of DspE is required for fire blight disease development [27, 28, 37, 38]. Mutations affecting the T3S system that result in a loss-of-pathogenicity phenotype are consequently hypothesized to be attributed to decreased DspE translocation by *E. amylovora*. To determine if HrcU_NPTH_ and HrpP are involved in regulating DspE secretion, *E. amylovora* strains were transformed with pLRT201 to express a DspE-CyaA fusion protein and incubated in vitro in *hrp*-inducing minimal medium (HrpMM) used to mimic conditions of the plant apoplast [39]. Proteins were extracted 48 hpi and subjected to one-dimensional SDS-PAGE separation and western blot analysis using an anti-CyaA antibody. As predicted, an Ea1189 strain harboring native *hrcU* secreted DspE in vitro while Ea1189∆*hrcU* failed to secrete any DspE protein (Fig 5a). Likewise, an Ea1189 strain synthesizing HrcU_N266A_ and Ea1189∆*hrpP* were also unable to secrete DspE (Fig 5a). Using SDS-PAGE analysis, we also show that Ea1189∆*hrcU* complemented with the full-length hrcU on pRRM1 secreted the native DspE protein, while Ea1189∆*hrcU* complemented with *hrcU*_N266A_ on pRRM2 was unable to secrete DspE (Fig 5b). Finally, the wild-type Ea1189 strain containing pRRM2 could still secrete DspE, indicating that HrcU_N266A_ does not exhibit a dominant-negative effect on HrcU (Fig 5b). These results show that HrcU_NPTH_ and HrpP are required for DspE secretion in vitro and suggest that Ea1189∆*hrpP* and Ea1189∆*hrcU*/pRRM2 are nonpathogenic due to loss of DspE secretion and translocation capability.

## Discussion

In this study the roles of HrcU and HrpP in regulating the T3S system in *E. amylovora* Ea1189 were explored. Using site-directed mutagenesis, phenotypic analyses, Y2H assays and protein visualization, HrcU and HrpP were shown to interact and mediate host-microbe interactions via the regulation of T3S system substrates like the effector DspE.

HrcU and HrpP were both confirmed to be pathogenicity factors in *E. amylovora* Ea1189. Ea1189∆*hrcU* and Ea1189∆*hrpP* were both unable to cause disease in immature pear fruits (Fig. 2a and 3c). Likewise, Ea1189∆*hrcU* and Ea1189∆*hrpP* were also unable to elicit a HR after inoculation into *N. benthamiana* (Fig 2b and 3c). These results are in agreement with previous observations regarding HrcU in *P. syringae* and *X. campestris* [5, 40]. Interestingly, while HrpP in *E. amylovora* and *P. syringae* are both required for disease and HR induction, the T3S4 homolog HpaC is not a pathogenicity factor in *X. campestris* [41, 42].

The important influence of HrcU and HrpP in facilitating disease development is hypothesized to stem from roles in regulating T3S hierarchy. In YscU/FlhB proteins, the regulation of T3S hierarchy hinges on a conserved NPTH amino acid motif [4, 5, 9, 13]. The cytoplasmic C-terminus of HrcU in *E. amylovora* encodes an NPTH motif (Fig. 1b). Notably, a site-directed mutation of *hrcU* resulting in the construct HrcU_N266A_ was unable to complement Ea1189∆*hrcU* suggesting that the role of HrcU in mediating plant-microbe interactions requires the presence of an asparagine residue at position 266 (Fig. 2). Ea1189∆*hrcU* strains expressing HrcU_N266A_ were nonpathogenic and here we report via western blot analysis that HrcU-mediated secretion of DspE was dependent on the integrity of its conserved NPTH motif (Fig. 5). While full-length *hrcU* was able to complement Ea1189∆*hrcU in trans* and restore DspE secretion, *hrcU*_N266A_ failed to rescue Ea1189∆*hrcU* mutant phenotypes (Fig. 2 and 5). These phenotypes are likely linked as DspE secretion is required for disease development [38]. Collectively, results illustrating the roles of HrcU and HrcU_N266A_ in *E. amylovora* reinforce data highlighting the importance of the NPTH motif in YscU/FlhB proteins as synonymous mutations in *X. campestris*, enteropathogenic *E. coli* and *Y. enterocolitica* also abolish disease development [5, 43, 44].

Notably though, while the NPTH motif is required for T3S-dependent disease development, YscU/FlhB mediated regulation of T3S differs between bacterial species. In *Salmonella*, the flagellar protein FlhB functions to establish hook assembly prior to filament secretion. Consequently, FlhB_N269A_ mutants fail to terminate hook protein secretion and initiate export of flagellin [43]. YscU in turn regulates the secretion of late substrates including translocators and effectors [4, 45].

One of the lesser-described proteins in the YscU/FlhB family is EscU from *Escherichia coli* EPEC strain E2348/69. EscU is particularly relevant to discussions of *E. amylovora*. Thomassin et al. [46] observed that EscU_N262A_ poorly secretes effectors while differentially regulating the secretion of effector chaperones. While chaperone EspC was secreted at wild-type levels, EspA, EspB and Tir were poorly secreted in vitro. The authors also revealed that Tir-induced actin polymeration was comparably reduced in infected HeLa cells. *E. amylovora* also utilizes a large consortium of chaperones to regulate effector secretion. Special attention should be given to how chaperones interact with HrcU to regulate secretion hierarchy [38, 47].

HrcU from *X. campestris* pv. *vesicatoria* represents the most characterized YscU/FlhB protein in plant pathogenic bacteria. Like YscU, HrcU_*Xcv*_ inhibits the secretion of late substrates. HrcU_*Xcv*_ NPTH mutants in turn over-secrete early T3S substrates analogous to increased hook secretion exhibited by FlhB flagellar mutants [5, 9]. Research concerning HrcU_Xcv_ has developed to reveal that while the NPTH motif in YscU/FlhB proteins has been the focus of much attention, additional HrcU domains and amino acid residues play a role in regulating T3S. While this research is the first demonstration of HrcU_*Ea*_ controlling substrate specificity in *hrp*1-T3S systems via the NPTH motif, more analyses are required to understand the full scope of HrcU-mediated T3S regulation.

Examinations of the T3S4 protein HrpP in *E. amylovora* revealed that, while exhibiting a c-terminal P-X-L-G motif, HrpP is diminutive like T3S4 proteins in other plant pathogenic bacteria. Conversely, mammalian bacterial pathogens exhibit T3S4 proteins up 3X in length. While structurally distinct, all T3S4 proteins share some commonalities. Here we present for the first time that HrpP is a pathogenicity factor in *E. amylovora* Ea1189. Ea1189∆*hrpP* was unable to generate disease development on immature pear fruit or induce a HR in *N. benthamiana* (Fig. 3c). Like HrcU_N266A_, Ea1189∆*hrpP* was also unable to secrete the T3S effector DspE as displayed using an in vitro western blot (Fig. 5).

Other T3S4 proteins, similarily to HrpP in *E. amylovora*, function to promote the secretion of late T3S substrates. Null mutations in YscP from *Y. enterocolitica*, HpaC from *X. campestris* and HrpP from *P. syringae* all fail to secrete late T3S substrates [16, 23, 41, 42]. More notably though, many T3S4 proteins also function to actively suppress the secretion of early T3S substrates and null mutations result in increased secretion of pilus subunits and inner rode proteins. FliK from the flagellum suppresses the secretion of the inner rod-like hook protein FlhB [13, 48]. Mutations in YscP trigger hyper-secretion of the pilin protein YscF and the inner rod protein YscI while HpaC mutants secrete more inner rod protein HrpB2 than wild-type [16, 23, 45].

Conversely, HrpP from *P. syringae* pv. *tomato* appears to function atypically relative to other know T3S4 proteins. While previously-described T3S4 proteins actively suppress early T3S events, Pst∆*hrpP* poorly secretes the early substrate HrpA, a T3S pilus subunit protein [42]. Consequently, HrpP_*Pst*_ may be more accurately described as a post-translational activator of T3S as opposed to a T3S4 protein. More experimentation will be required to determine if HrpP_*Ea*_ also functions as a post-translation activator though it is important to note that HrpP_*Pst*_ has not been observed to interact with HrcU_*Pst*_ while evidence suggests that HrpP_*Ea*_ binds HrcU_*Ea*_ as has been reported for other canonical T3S4 proteins [25, 49–52].

Using Y2H analysis to explore protein-protein interactions, we demonstrate that HrpP and the cytoplasmic tail of HrcU bind when co-expressed in *Saccharomyces cerevisiae* (Fig. 4). These results conform to previous observations in other plant and animal pathogenic bacteria. For example, in *Salmonella*, FlhB binds directly to FliK [50] and in *Xanthomonas*, HrcU_Xcv_ directly binds HpaC [5, 9]. Noteably, YscU from *Yersinia* has never been shown to interact with YscP indicating potential variability in T3S hierarchy regulation [17]. Despite some variability, to date, all known YscU/FlhB_NPTH_ domains are required for T3S function and disease development.

In *E. amylovora*, virulence and DspE secretion assays are consistent with previous observations concerning the functional importance of the HrcU_NPTH_ domain (Fig. 2 and 5). Our Y2H results reveal however that while HrcU_NPTH_ affects HrpP_*Ea*_ interactions, the domain is not required for binding (Fig. 4). Confirmation of observed HrcU_*Ea*_-HrpP_*Ea*_ interaction data in yeast will require future use of more sensitive and specific techniques such as co-immunoprecipitation assays. In addition, work by Haunser and Buttner [9] also indicates that HrcU_*Xcv*_ exhibits multiple amino acid residues, in addition to the NPTH domain, with functional significance for plant disease outcomes and, in response, a more through mutational analysis will be required to understand the role of HrcU_*Ea*_ in T3S regulation and interactions with HrpP_*Ea*_. Considering that both HrcU and HrpP are pathogenicity factors in *E. amylovora* and as both Ea1189∆*hrcU* and Ea1189∆*hrpP* exhibit impaired DspE secretion, it is tempting to speculate that interactions between HrcU and HrpP may be important for their relative roles in pathogenicity.

## Conclusions

Here we report the first information regarding the roles of HrcU and HrpP in regulating T3S of DspE in *E. amylovora*. Both proteins were shown to be required for pathogenesis in *E. amylovora* Ea1189, were required for DspE secretion, and show evidence of inter-protein interactions. Future work should focus on how HrcU and HrpP regulate the secretion of the inner rod protein HrpJ and the needle protein HrpA as well as how regulator copy number influences HrpA secretion. In addition, as effector chaperones are known to be regulated by YscU/FlhB proteins in animal pathogenic bacteria, identifying a role for HrcU in regulating plant pathogenic effector chaperones would be a novel contribution to the plant pathology community.

## Methods

### Bacterial strains and growth conditions

Table 2 lists bacterial strains and plasmids used in this study. Unless otherwise referenced, bacterial strains were grown in Luria Bertani (LB) broth supplemented with 50 μg ml^−1^ ampicillin, 20 μg ml^−1^ chloramphenicol, 12 μg ml^−1^ oxytetracycline or 30 μg ml^−1^ kanamyacin where appropriate. All strains were cultured at 28 °C in a shaking incubator.

**Table 2:** Bacterial strains and plasmids and their relevant characteristics

Strains & Plasmids	Relevant characteristics^a^	Source or reference
*Escherichia coli* strain		
DH5α	F- 80dlacZ, ΔM15, Δ(lacZYA-argF)U169, endA1, recA1, hsdR17(rK–mK+), deoR, thi-1, supE44, gyrA96, relA1 λ-	Invitrogen, CA, USA
Yeast strain		
*Saccharomyces cerevisiae* AH109	MATa, trp1-901, leu2-3, 112, ura3-52, his3-200, gal4Δ, gal80Δ, LYS2 : : GAL1_UAS_-GAL1_TATA_-HIS3, GAL2_UAS_-GAL2_TATA_-ADE2, URA3 : : MEL1_UAS_-MEL1_TATA_-lacZ	[61]
*Erwinia amylovora* strains		
Ea1189	Wild type	[62]
Ea1189∆*hrcU*	*hrcU* deletion mutant, Cm^R^	This study
Ea1189∆*hrpP*	*hrpP* deletion mutant, Cm^R^	This study
Plasmids		
pBBR1-MCS3	Tc^R^, broad host-range cloning vector	[54]
pGADT7	LEU2, Amp^R^, Y2H activation vector	Clontech, CA, USA
pGBKT7	TRP1, Km^R^, Y2H bait vector	Clontech, CA, USA
pLRT201	Amp^R^, pMJH20 expressing DspE(1-737)-CyaA	[38]
pMJH20	Amp^R^, pWSK29 containing codons 2 to 406 of CyaA	[63]
pRRM1	Tc^R^, pBBR1-MCS3 containing *hrcU*	This study
pRRM2	Tc^R^, pBBR1-MCS3 containing *hrcU*_N266A_	This study
pRRM3	Amp^R^, pGADT7 containing *hrpP*	This study
pRRM4	Amp^R^, pGADT7 containing *hrpJ*	This study
pRRM5	Km^R^, pGBKT7 containing *hrcU*	This study
pRRM6	Km^R^, pGBKT7 containing *hrcU*_N266A_	This study
pRRM7	Km^R^, pGBKT7 containing *hrcU*_209-360_	This study
pRRM8	Km^R^, pGBKT7 containing *hrcU*_209-360, N266A_	This study
pRRM9	Tc^R^, pAlter-Ex1 containing *hrcU*	This study
pRRM10	Amp^R^, pAlter-Ex1 containing *hrcU*_N266A_	This study
pAlter-Ex1	Tc^R^, mutagenesis vector	Promega, WI, USA
pKD3	Amp^R^, Cm^R^ mutagenesis cassette template	[59]
pKD46	Amp^R^, expresses λ red recombinase	[59]

^a^Cm^R^, Tc^R^, Amp^R^, Km^R^ indicates resistance to chloramphenicol, oxytetracycline, ampicillin and kanamycin

### DNA manipulation and cloning

Restriction enzyme digestion, T4 DNA ligation, and PCR amplification of genes were carried out using standard molecular techniques [53]. DNA extraction, PCR purification, plasmid extraction, and isolation of DNA fragments from agarose were performed with related kits (Qiagen, Valencia, CA). The sequences of oligonucleotide primers used in this study are listed in Additional file 1: Table S1. All DNA was sequenced at the Research Technology Support Facility at Michigan State University. Double digestion and directional ligation into pBBR1MCS3 [54] with PCR-generated gene sequences was utilized for mutant strain complementation. Final constructs were transformed into competent Ea1189 by electroporation and screened on LB agar plates amended with oxytetracycline.

### Bioinformatics

Lasergene® 7.2.0 software suite was used to manage nucleic and amino acid sequences (DNASTAR, Madison, WI). Genes were annotated in agreement with the *E. amylovora* ATCC 49946 genome [55]. Protein sequence conservation was determined using BLAST programs at NCBI (http://blast.ncbi.nlm.nih.gov/Blast.cgi) [56]. The sequences of T3S4 domain-containing proteins were acquired from NCBI, with the accession numbers: ACI16082.1 (*Yersinia enterocolitica* YscP), CDH77977.1 (*Pseudomonas aeruginosa* PscP), WP_012228919.1 (*Y. pestis* FliK), GAO95686.1 (*P. syringae* HrpP), WP_020830096.1 (*Ralstonia solanacearum* YscP), and WP_004155345.1 (*E. amylovora* HrpP). The T3S4 domains were identified using the T3S4 AA sequences described from a previous work [21]. Putative transmembrane domains were predicted using the DAS - Transmembrane Prediction server (http://www.sbc.su.se/~miklos/DAS) [31]. T-Coffee multiple sequence alignment software (http://www.tcoffee.org/homepage.html) was used to create all amino acid sequence alignments [32]. Multiple sequence alignments were visualized using Weblogo 2.8.2 (http://weblogo.berkeley.edu) [57].

### Virulence and hypersensitive response assays

The virulence of *E. amylovora* Ea1189 was assayed using a standard immature pear fruit assay as described previously [58]. In brief, bacterial strains were cultured overnight, washed, and resuspended in 0.5x phosphate buffered saline (PBS) to 1 × 10^3^ to 1 × 10^4^ CFU/ml. Immature pear fruits (*Pyrus communis* L. cv. Bartlett) were surface sterilized with 10 % bleach, dried in laminar flow hood, and pricked with a needle prior to application of 2 μl bacterial suspension. Inoculated pears were incubated at 28 °C in humidified chambers. Symptoms were recorded 6 days post inoculation. The experiments were repeated three times with six replications per experiment. To study elicitation of the HR during incompatible interactions, *E. amylovora* strains were cultured over night in LB broth. Bacterial cells were collected via centrifugation and washed twice with 0.5X PBS. Cells were resuspended and adjusted to a final concentration of 1 × 10^7^ CFU ml^−1^ in 0.5X PBS. 100 μl of cell suspension were in turn infiltrated into 9-week-old N. benthamiana leaves using a syringe and HR was observed 16 hpi.

### Mutagenesis

*E. amylovora* site-directed nonpolar chromosomal mutants were generated using the phage λ Red recombinase system previously described [59]. Briefly, *E. amylovora* strain Ea1189 harboring pKD46, encoding recombinases red β, γ, and exo, was cultured overnight at 28 °C in a shaking incubator. Strains were reinoculated with 0.1 % L-arabinose in LB broth and cultured for four hours to exponential phase. Cells were made electrocompetent and stored at -80 °C. Homologous recombination fragments encoding acetyltransferase cassettes were generated via polymerase chain reaction (PCR) using the plasmid pKD3 as a template. A PCR purification kit (Qiagen; Valencia, CA) was using to purify recombination fragments before electroporation into competent Ea1189. LB agar amended with chloramphenicol was used to screen putative mutants and single-gene recombinatorial deletion was confirmed using PCR and functional complementation.

Site-directed point-mutations were introducted into HrcU using and as described by the Altered Sites® II in vitro Mutagenesis System (Promega; Madison, WI). Briefly, full-length HrcU (NC_013971) was cloned into pAlter-Ex1 via NcoI and NsiI restriction sites creating pRRM9. Mutagenic oligonucleotide HrcU_N266A (5′-GACCTGCTGCTGGTCGCTCCCACGCACTATGCG-3′) was designed to convert the asparagine amino acid at position 266 to an alanine residue. pRRM9 and HrcU_N266A primer were denatured and phosphorylated respectively and via PCR pAlter-Ex1(*hrcU*_N266A_) (pRRM10) was synthesized and transformed into competent *E. coli* cells.

### Yeast two-hybridization

The bait vector pGBKT7 and the prey vector pGADT7 were used for yeast expression and Y2H screening (Clontech, Mountain View, CA, USA). A Frozen-EZ Yeast Transformation II Kit was used to create competent *Saccharomyces cerevisiae* AH109 and for cotransformation of bait and prey (Zymo Research Corporation, Orange, CA, USA). Transformants were selected on minimal SD agar amended with -Ade/-His/-Leu/-Trp dropout supplement and Mel1 α-galactosidase activity was detected using topically applied X-α-Gal at 4 ug ul^−1^ (Clontech, Mountain View, CA, USA). The intensity of blue color was quantified using ImageJ (http://imagej.nih.gov/ij/download.html). Kendall rank correlation coefficient τ tests were performed to determine the statistical significance. Equations are listed below:

**Table:** 

$\uptau =\underline {{\mathrm{n}}_{\mathrm{c}}-{\mathrm{n}}_{\mathrm{d}}}$	n represents sample size
1/2n(n − 1)	n_c_ is the number of concordant pairs
$\mathrm{z}=\underline {3*\tau\ *\surd \mathrm{n}\left(\mathrm{n}-1\right)}$	n_d_ is the number of discordant pairs
√ 2(2n + 5)	

### Secretion assays

Strains were cultured overnight in 50 ml LB broth at 28 °C. Cells were washed twice with 0.5X PBS, and resuspended in 50 ml minimal medium, pH 5.7 [60]. Strains were induced for 48 h with shaking, collected by centrifugation, and the supernatant was filtered using 0.22 μm vacuum filtration (Millipore, Billerica, MA, USA). Filtrate was supplemented with 0.5 mM phenylmethylsulfonyl fluoride and concentrated to approximately 500 μl using 10-kDa Amicon centrifugal filter units (Millipore, Billerica, MA, USA). For ease of detection of secreted DspE protein, we used plasmid pLRT201 which is an expression construct that encodes the first 737 amino acids of DspE fused to an adenylate cyclase (CyaA) reporter [38]. We have previously demonstrated secretion of this DspE_1-737_-CyaA fusion protein via the T3S system [38]. DspE secretion was examined in the WT *E. amylovora* Ea1189/pLRT201, Ea1189∆*hrcU*/pLRT201, Ea1189∆*hrpP*/pLRT201 and Ea1189∆*hrcU*/pRRM2/pLRT201.

For western blot analysis, proteins were analyzed using anti-CyaA antibody (Santa Cruz Biotechnology, Santa Cruz, CA). For protein visualization, proteins were additionally purified to remove biofilm polysaccharides as previously described [29]. Briefly, protein samples were extracted twice with 0.5 volume of water-saturated phenol and precipitated with by the addition of 5 volumes 100 mM ammonium acetate in methanol. After overnight incubated at −20 °C, protein were extracted via centrifugation, resuspended in 50 ul water and reprecipitated in 500 μl of cold acetone. Samples were again incubated overnight at −20 °C and protein pellets were collected by centrifugation at 13,000 g at 4 °C for 30 min and subsequent resuspension in 50 μl 5 % acetic acid supplement with 0.5 mM PMSF. A bicinchoninic acid (BCA) protein assay kit was used to measure protein concentrations and concentrations were adjusted to 1 μg μl^−1^. Eight μg of each protein sample were used for western blot analysis.

### Ethics approval and consent to participate

Not applicable.

### Consent for publication

Not applicable.

### Availability of data and materials

All data in support of our findings is contained within this manuscript or included as supplemental figures.

## Additional file

Additional file 1:
**Table S1.** Description of data: Sequences of oligonucleotide primers used in this study. (DOCX 109 kb)

## Abbreviations

HR: Hypersensitive response
Hrc: Hypersensitive response and conserved
Hrp: Hypersensitive response and pathogenicity
NPTH: Asparigine-proline-threonine-histidine domain found in YscU/FlhB proteins
T3S: Type III secretion
T3S4: Type III secretion substrate specificity switch

## References

[1] Galan JE, Collmer A (1999). Type III secretion machines: bacterial devices for protein delivery into host cells. Science.

[2] Williams AW, Yamaguchi S, Togashi F, Aizawa SI, Kawagishi I, Macnab RM (1996). Mutations in *fliK* and *flhB* affecting flagellar hook and filament assembly in *Salmonella typhimurium*. J Bacteriol.

[3] Minamino T, Macnab RM (2000). Domain structure of *Salmonella* FlhB, a flagellar export component responsible for substrate specificity switching. J Bacteriol.

[4] Sorg I, Wagner S, Amstutz M, Muller SA, Broz P, Lussi Y, Engel1 A, Cornelis GR. YscU recognizes translocators as export substrates of the *Yersinia* injectisome. EMBO J. 2007;26:3015–24. 10.1038/sj.emboj.7601731 PMC1894769 17510628

[5] Lorenz C, Buttner D (2011). Secretion of early and late substrates of the type III secretion system from *Xanthomonas* is controlled by HpaC and the C-terminal domain of HrcU. Mol Microbiol.

[6] Hartmann N, Schulz S, Lorenz C, Fraas S, Hause G, Buttner D (2014). Characterization of HrpB2 from *Xanthomonas campestris* pv. *vesicatoria* identifies protein regions that are essential for type III secretion pilus formation. Microbiology.

[7] Lohou D, Turner M, Lonjon F, Cazale AC, Peeters N, Genin S, Vailleau F (2014). HpaP modulates type III effector secretion in *Ralstonia solanacearum* and harbours a substrate specificity switch domain essential for virulence. Mol Plant Pathol.

[8] Buttner D (2012). Protein export according to schedule: architecture, assembly, and regulation of type III secretion systems from plant- and animal-pathogenic bacteria. Microbiol Mol Biol Rev.

[9] Hausner J, Buttner D (2014). The YscU/FlhB homologue HrcU from *Xanthomonas* controls type III secretion and translocation of early and late substrates. Microbiology.

[10] Berger C, Robin GP, Bonas U, Koebnik R (2010). Membrane topology of conserved components of the type III secretion system from the plant pathogen Xanthomonas campestris pv. vesicatoria. Microbiology.

[11] Diepold A, Wiesand U, Amstutz M, Cornelis GR (2012). Assembly of the *Yersinia* injectisome: the missing pieces. Mol Microbiol.

[12] Allaoui A, Woestyn S, Sluiters C, Cornelis GR (1994). YscU, a *Yersinia enterocolitica* inner membrane protein involved in Yop secretion. J Bacteriol.

[13] Hirano T, Yamaguchi S, Oosawa K, Aizawa SI (1994). Roles of FliK and FlhB in determination of flagellar hook length in *Salmonella typhimunum*. J Bacteriol.

[14] Macnab RM (2000). Action at a distance - bacterial flagellar assembly. Science.

[15] Ferris HU, Furukawa Y, Minamino T, Kroetz MB, Kihara M, Namba K, Macnab RM (2005). Protein synthesis, post-translation modification, and degradation: FlhB regulates ordered export of flagellar components via autocleavage mechanism. J Biol Chem.

[16] Lorenz C, Wolsch T, Rossier O, Bonas U, Buttner D (2008). HpaC Controls Substrate Specificity of the *Xanthomonas* Type III Secretion System. PLoS Pathog.

[17] Riordan KE, Schneewind O (2008). YscU cleavage and the assembly of *Yersinia* type III secretion machine complexes. Mol Microbiol.

[18] Lorenz C, Buttner D (2009). Functional characterization of the type III secretion ATPase HrcN from the plant pathogen *Xanthomonas campestris* pv. *vesicatoria*. J Bacteriol.

[19] Lorenz C, Hausner J, Buttner D (2012). HrcQ provides a docking site for early and late type III secretion substrates from *Xanthomonas*. PLoS One.

[20] Hartmann N, Buttner D (2013). The inner membrane protein HrcV from *Xanthomonas* is involved in substrate docking during type III secretion. Mol Plant Microbe Interact.

[21] Agrain C, Callebaut I, Journet L, Sorg I, Paroz C, Mota LJ, Cornelis GR (2005). Characterization of a Type III secretion substrate specificity switch (T3S4) domain in YscP from *Yersinia enterocolitica*. Mol Microbiol.

[22] Minamino T, Gonzalez-Pedrajo B, Yamaguchi K, Aizawa SI, Macnab RM (1999). FliK, the protein responsible for flagellar hook length control in *Salmonella*, is exported during hook assembly. Mol Microbiol.

[23] Edqvist JP, Olsson J, Lavander M, Sundberg L, Forsberg A, Wolf-Watz H, Lloyd SA (2003). YscP and YscU regulate substrate specificity of the *Yersinia* type III secretion system. J Bacteriol.

[24] Journet L, Agrain C, Broz P, Cornelis GR (2003). The needle length of bacterial injectisomes is determined by a molecular ruler. Science.

[25] Schulz S, Buttner D (2011). Functional characterization of the type III secretion substrate specificity switch protein HpaC from *Xanthomonas campestris* pv. *vesicatoria*. Infect Immun.

[26] Malnoy M, Martens S, Norelli JL, Barny M-A, Sundin GW, Smiths THM, Duffy B (2012). Fire blight: applied genomc insights of the pathogen and host. Annu Rev Phytopathol.

[27] Barny MA, Guinebretiere MH, Marcais B, Coissac E, Paulin JP, Laurent J (1990). Cloning of a large gene cluster involved in *Erwinia amylovora* CFBP1430 virulence. Mol Microbiol.

[28] Bauer DW, Beer SV (1991). Further characterization of an hrp gene cluster *Erwinia amylovora*. Mol Plant Microbe Interact.

[29] Nissinen RM, Ytterberg AJ, Bogdanove AJ, van Wijk KJ, Beer SV (2007). Analyses of the secretomes of *Erwinia amylovora* and selected hrp mutants reveal novel type III secreted proteins and an effect of HrpJ on extracellular harpin levels. Mol Plant Pathol.

[30] Lavander M, Sundberg L, Edqvist PJ, Lloyd SA, Wolf-Watz H, Forsberg A (2002). Proteolytic cleavage of the FlhB homologue YscU of *Yersinia pseudotuberculosis* is essential for bacterial survival but not for type III secretion. J Bacteriol.

[31] Cserzo M, Wallin E, Simon I, von Heijne G, Elofsson A (1997). Prediction of transmembrane α-helices in prokaryotic membrane proteins: the dense alignment surface method. Protein Eng.

[32] Notredame C, Higgins DG, Heringa J (2000). T-Coffee: A novel method for fast and accurate multiple sequence alignment. J Mol Biol.

[33] Heckman KL, Pease LR (2007). Gene splicing and mutagenesis by PCR-driven overlap extension. Nature Protoc.

[34] Steinberger EM, Beer SV (1988). Creation and complementation of pathogenicity mutants of *Erwinia amylovora*. Mol Plant Microbe Interact.

[35] Deane JE, Abrusci P, Johnson S, Lea SM (2010). Timing is everything: the regulation of type III secretion. Cell Mol Life Sci.

[36] Ji H, Dong H (2015). Key steps in type III secretion system (T3SS) towards translocon assembly with potential sensor at plant plasma membrane. Mol Plant Pathol.

[37] Bocsanczy AM, Nissinen RM, Oh C-S, Beer SV (2008). HrpN of *Erwinia amylovora* functions in the translocation of DspA/E into plant cells. Mol Plant Pathol.

[38] Triplett LR, Melotto M, Sundin GW (2009). Functional analysis of the N terminus of the *Erwinia amylovora* secreted effector DspA/E reveals features required for secretion, translocation, and binding to the chaperone DspB/F. Mol Plant Microbe Interact.

[39] Wei ZM, Laby RJ, Zumoff CH, Bauer DW, He SY, Collmer A, Beer SV (1992). Harpin, elicitor of the hypersensitive response produced by the plant pathogen *Erwinia amylovora*. Science.

[40] Charkowski AO, Huang HC, Collmer A (1997). Altered localization of HrpZ in *Pseudomonas syringae* pv. *syringae hrp* mutants suggests that different components of the type III secretion pathway control protein translocation across the inner and outer membranes of Gram-negative bacteria. J Bacteriol.

[41] Buttner D, Lorenz C, Weber E, Bonas U (2006). Targeting of two effector protein classes to the type III secretion system by a HpaC- and HpaB-dependent protein complex from *Xanthomonas campestris* pv. *vesicatoria*. Mol Microbiol.

[42] Morello JE, Collmer A (2009). *Pseudomonas syringae* HrpP is a type III secretion substrate specificity switch domain protein that is translocated into plant cells but functions atypically for a substrate-switching protein. J Bacteriol.

[43] Fraser GM, Hirano T, Ferris HU, Devgan LL, Kihara M, Macnab RM (2003). Substrate specificity of type III flagellar protein export in *Salmonella* is controlled by subdomain interactions in FlhB. Mol Microbiol.

[44] Zarivach R, Deng W, Vuckovic M, Felise HB, Nguyen HV, Miller SI, Finlay BB, Strynadka NCJ (2008). Structural analysis of the essential self-cleaving type III secretion proteins EscU and SpaS. Nature.

[45] Wood SE, Jin J, Lloyd SA (2008). YscP and YscU switch the substrate specificity of the *Yersinia* type III secretion system by regulating export of the inner rod protein YscI. J Bacteriol.

[46] Thomassin JL, He X, Thomas NA (2011). Role of EscU auto-cleavage in promoting type III effector translocation into host cells by enteropathogenic *Escherichia coli*. BMC Microbiol.

[47] Triplett LR, Wedemeyer WJ, Sundin GW (2010). Homology-based modeling of the *Erwinia amylovora* type III secretion chaperone DspF used to identify amino acids required for virulence and interaction with the effector DspE. Res Microbiol.

[48] Minamino T, Moriya N, Hirano T, Hughes KT, Namba K (2009). Interaction of FliK with the bacterial flagellar hook is required for efficient export specificity switching. Mol Microbiol.

[49] Botteaux A, Sani M, Kayath CA, Boekema EJ, Allaoui A (2008). Spa32 interaction with the inner-membrane Spa40 component of the type III secretion system of *Shigella flexneri* is required for the control of the needle length by a molecular tape measure mechanism. Mol Microbiol.

[50] Morris DP, Roush ED, Thompson JW, Moseley MA, Murphy JW, McMurry JL (2010). Kinetic characterization of *Salmonella* FliK-FlhB interactions demonstrates complexity of the type III secretion substrate-specificity switch. Biochemistry.

[51] Diepold A, Wagner S (2014). Assembly of the bacterial type III secretion machinery. FEMS Microbiol Rev.

[52] Marlovits TC, Kubori T, Lara-Tejero M, Thomas D, Unger VM, Galan JE (2006). Assembly of the inner rod determines needle length in the type III secretion injectisome. Nature.

[53] Sambrook JF, Fritsch EF, Maniatis TP (1989). Molecular cloning: a laboratory manual.

[54] Kovach ME, Elzer PH, Hill DS, Robertson GT, Farris MA, Roop RM, Peterson KM (1995). Four new derivatives of the broad-host-range cloning vector pBBR1MCS, carrying different antibiotic-resistance cassettes. Gene.

[55] Sebaihia M, Bocsanczy AM, Biehl BS, Quail MA, Perna NT, Glasner JD, DeClerck GA, Cartinhour S, Schneider DJ, Bentley SD, Parkhill J, Beer SV (2010). Complete genome sequence of the plant pathogen *Erwinia amylovora* strain ATCC 49946. J Bacteriol.

[56] Altschul SF, Madden TL, Schaffer AA, Zhang J, Zhang Z, Miller W, Lipman DJ (1997). Gapped BLAST and PSI-BLAST: a new generation of protein database search programs. Nucleic Acids Res.

[57] Crooks GE, Hon G, Chandonia JM, Brenner SE (2004). WebLogo: a sequence logo Generator. Genome Res.

[58] Zhao Y, Blumer SE, Sundin GW (2005). Identification of *Erwinia amylovora* genes induced during infection of immature pear tissue. J Bacteriol.

[59] Datsenko KA, Wanner BL (2000). One step inactivation of chromosomal genes in *Escherichia coli* K-12 using PCR products. Proc Natl Acad Sci U S A.

[60] Huynh TV, Dahlbeck D, Staskawicz BJ (1989). Bacterial blight of soybean: regulation of a pathogen gene determining host cultivar specificity. Science.

[61] James P, Halladay J, Craig EA (1996). Genomic libraries and a host strain designed for highly efficient two-hybrid selection in yeast. Genetics.

[62] Burse A, Weingart H, Ullrich MS (2004). The phytoalexin-inducible multidrug efflux pump AcrAB contributes to virulence in the fire blight pathogen *Erwinia amylovora*. Mol Plant Microbe Interact.

[63] Miao EA, Miller SI (2000). A conserved amino acid sequence directing intracellular type III secretion by *Salmonella typhimurium*. Proc Natl Acad Sci U S A.

