# Supplementary material for: HrcU and HrpP are pathogenicity factors in the fire blight pathogen Erwinia amylovora required for the type III secretion of DspA/E
Source: BMC Microbiol. 2016 May 20;16:88. doi: 10.1186/s12866-016-0702-y (PMC4875606; doi:10.1186/s12866-016-0702-y)
Supplement: Additional file 1: — Table S1. Description of data: Sequences of oligonucleotide primers used in this study. (DOCX 109 kb) [file 12866_2016_702_MOESM1_ESM.docx]

| **Table S1** Oligonucleotide primers utilized in this study | |
| --- | --- |
| Name | Sequence |
| HrcUKO.F | TTGGCAGAAAAAACGGAAAAACCCACGGCGAAAAAGCTACAGGATGCGCGGTGTAGGCTGGAGCTGCTTC |
| HrcUKO.R | TTATTCGGCTTCCAGTTCGATCACCTCATCGGTTATCTGGTCTTCCAGCTCATATGAATATCCTCCTTA |
| HrpPKO.F | ATGAATACATCGGGTTATTCTGACCGGCTGCCGCCCTCCCCGCGCCAAACGTGTAGGCTGGAGCTGCTTC |
| HrpPKO.R | TCATGGCGCTTCTCCTTGCTCTATCGACAACCGTACCGGGCCGTTGAGCGCATATGAATATCCTCCTTA |
| HrcU_N266A | GACCTGCTGCTGGTCGCTCCCACGCACTATGCG |
| hrcU(NcoI).F | TGACCCATGGTTATTCGGCTTCCAGTTCG |
| hrcU(NsiI).R | CATGATGCATGGCAGAAAAAACGGAAAAACCCAC |
| Tc_KO | GCCGGGCCTCTTGCGGGCGTCCATTCC |
| Amp_RP | GTTGCCATTGCTGCAGGCATCGTGGTG |
| hrpJ(EcoRI).F | TGCAGAATTCAAAATTGCTCCCGTTTTACC |
| hrpJ(BamHI).R | GCTAAACCCAGGACGGCGCTGTAAGGATCC |
| hrpP(NdeI).F | TTAGCATATGAATACATCGGGTTATTCTGACCGG |
| hrpP(ClaI).R | TAGTATCGATTCATGGCGCTTCTCCTTGCTC |
| hrcU(EcoRI).F | GAATTCTTGGCAGAAAAAACGGAAAAACCCACG |
| hrcU(SalI).R | GTCGACTTATTCGGCTTCCAGTTCGATCACCTC |
| hrcU_CT(EcoRI).F | TGCAGAATTCGTAGAAGAAGCCGACCTGCTGCTG |
| hrcU_CT(SalI).R | GACTGTCGACTTATTCGGCTTCCAGTTCGATCACCTC |
